# Genetic Diversity and Antimicrobial Resistance of *Escherichia coli* from Human and Animal Sources Uncovers Multiple Resistances from Human Sources

**DOI:** 10.1371/journal.pone.0020819

**Published:** 2011-06-08

**Authors:** A. Mark Ibekwe, Shelton E. Murinda, Alexandria K. Graves

**Affiliations:** 1 United States Salinity Laboratory, United States Department of Agriculture-Agriculture Research Service (USDA-ARS), Riverside, California, United States of America; 2 Department of Animal and Veterinary Sciences, California State Polytechnic University, Pomona, California, United States of America; 3 Department of Soil Sciences, North Carolina State University, Raleigh, North Carolina, United States of America; University of Sydney, Australia

## Abstract

*Escherichia coli* are widely used as indicators of fecal contamination, and in some cases to identify host sources of fecal contamination in surface water. Prevalence, genetic diversity and antimicrobial susceptibility were determined for 600 generic *E. coli* isolates obtained from surface water and sediment from creeks and channels along the middle Santa Ana River (MSAR) watershed of southern California, USA, after a 12 month study. Evaluation of *E. coli* populations along the creeks and channels showed that *E. coli* were more prevalent in sediment compared to surface water. *E. coli* populations were not significantly different (*P* = 0.05) between urban runoff sources and agricultural sources, however, *E. coli* genotypes determined by pulsed-field gel electrophoresis (PFGE) were less diverse in the agricultural sources than in urban runoff sources. PFGE also showed that *E. coli* populations in surface water were more diverse than in the sediment, suggesting isolates in sediment may be dominated by clonal populations.Twenty four percent (144 isolates) of the 600 isolates exhibited resistance to more than one antimicrobial agent. Most multiple resistances were associated with inputs from urban runoff and involved the antimicrobials rifampicin, tetracycline, and erythromycin. The occurrence of a greater number of *E. coli* with multiple antibiotic resistances from urban runoff sources than agricultural sources in this watershed provides useful evidence in planning strategies for water quality management and public health protection.

## Introduction

*E. coli* are widely used as indicators of fecal contamination of waterways in most urban areas. The organism naturally occurs in the intestinal tracts of warm-blooded animals [1], and is released into the environment through deposition of fecal material. In a typical mixed watershed, host sources of *E. coli* may be from humans, farm animals, wildlife, and pets, among others [2], [3]. These hosts are generally described as primary habitats, and until recently *E. coli* was believed to survive poorly in the environment, and not to grow in secondary habitats such as surface water, sediment, and soil [4], [5]. However, it has been shown that *E. coli* can survive in the secondary environments for long periods of time and grow in water, sediment, and soil even in temperate environments [2], [6], [7], [8], [9], [10], [11].

While *E. coli* has diverse genotypic and phenotypic characteristics, some characteristics are shared among strains exposed to similar environments due to selection pressure [5]. The level of selective pressure exerted in a mixed catchment area may be a useful criterion for identifying the host sources of *E. coli* in the watershed. One such tool to aid with examining the selection pressure on *E. coli* is assessing their antimicrobial sensitivities [12], [13]. There are at least 17 classes of antimicrobials approved for use in food animals in the United States [14]. These antimicrobials provide benefits such as improved animal health, higher productivity, and in some cases, reduction in foodborne pathogens [15], and other pathogens of public health significance. However, use of antibiotics for agricultural purposes, particularly for growth enhancement, has come under much scrutiny worldwide, as it has been shown to contribute to the increased prevalence of antibiotic-resistant bacteria of public health significance [15]. In 2003, the FDA directly addressed the issue of risks associated with use of antibiotics in food animals with the release of the Guidance for Industry 152 (www.fda.gov/cvm), which outlined steps for risk assessment in the evaluation of new animal drugs in terms of microbial food safety [16].

In the Santa Ana River watershed, there are about 200,000 cattle in a 77 km^2^ area and over 1.4 million human residents. The high numbers of concentrated animal feeding operations (CAFOs) and human population gives rise to a major concern relating tothe potential risk associated with the distribution, diversity, and antimicrobial resistance of *E. coli* isolates in surface water and sediment. *E. coli* may survive in surface water and sediment because of high nutrient content from manure originating from CAFOs, runoff from large residential areas, warm temperatures, and inputs from other urban sources. Currently, available data from the watershed demonstrates that both existing and Environmental Protection Agency (EPA)-recommended bacterial water quality criteria are routinely exceeded in the watersheds, often by one or more orders of magnitude [17], [18], [19].

This study was conducted to determine the frequency of occurrence of generic *E. coli* in the sediment and surface water of creeks and rivers within the middle Santa Ana River (MSAR) watershed, which may influence water quality and subsequently pose a risk to human health. Furthermore, we sought to characterize *E. coli* isolates obtained in terms of their genetic diversity using pulsed-field gel electrophoresis (PFGE). Finally, because of the paramount importance of presence of pharmaceutical and personal care products in receiving waters (which can affect growth of macro- and micro-organisms), antimicrobial resistance profiles for the *E. coli* isolates, and presence of specific genes that encode for antibiotic resistance, were determined. We hypothesized that antimicrobial sensitivity of *E. coli* from the sediments and surface waters of creeks associated with food animal production would be different from creeks that are associated with residential areas.

## Materials and Methods

### Ethics statement

Throughout this study, normal operational procedures of the forest service and state park on the creeks and channel were followed. Permits to enter the parks and channels were obtained from the regional parks.

### Study area and sample collection

**Figure 1:**
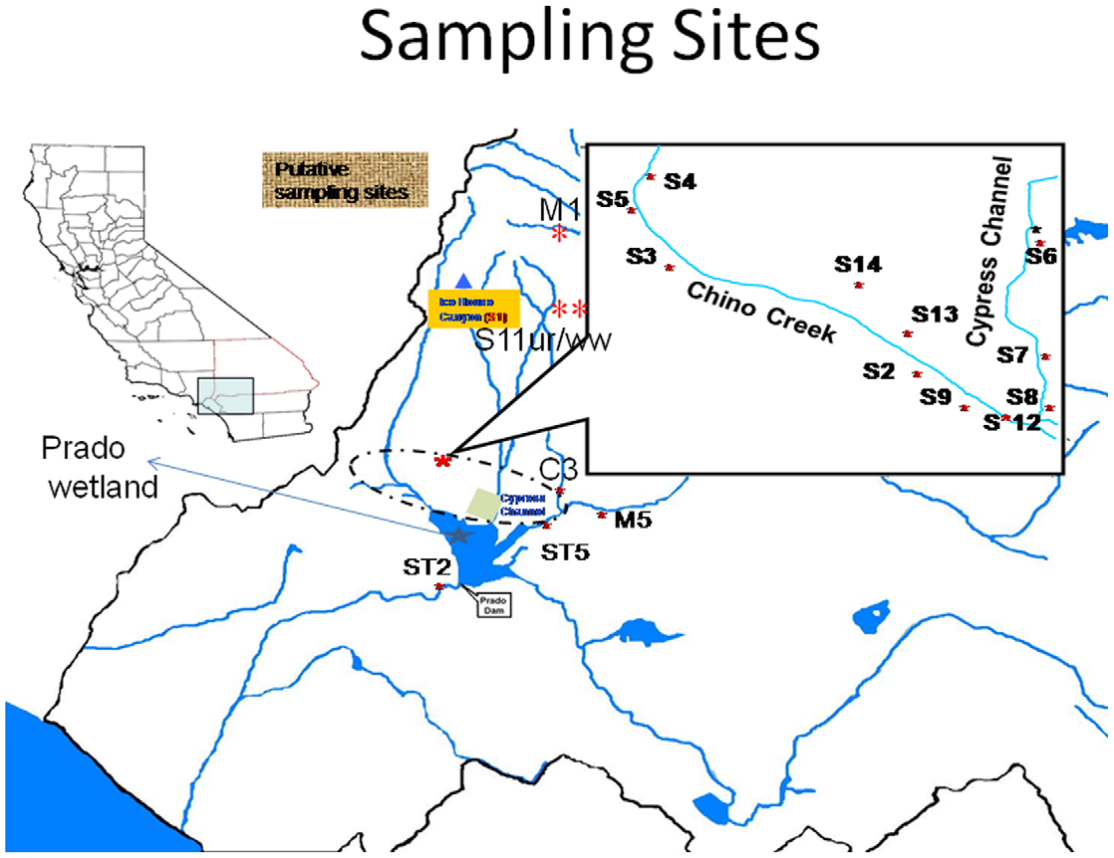
Sites used for the study along the MSAR watershed. Chino Creek and Cypress channel are the two main channels in the MSAR watershed with inputs from urban runoff and agricultural activities, respectively. Both creeks empty into the Santa Ana River.

This study was conducted in the middle Santa Ana River (MSAR) watershed area that covers ∼1,264 km^2^ and lies largely in the southwestern corner of San Bernardino County and the northwestern corner of Riverside County (Fig. 1). A small part of Los Angeles County (i.e., Pomona/Claremont area) is included. The current population of the watershed, based upon the 2000 census data, is ∼1.4 million people. Land use in the MSAR watershed varies between urban and agriculture. Although originally developed as an agricultural area, the watershed is rapidly urbanizing. Open space areas include the National Forest and State Park lands. The principal remaining agricultural area in the watershed was formerly referred to as the Chino Dairy Preserve. This area is located in the south central part of the Chino Basin sub watershed and contains approximately 200,000 cows in a 77 km^2^ area (although this number is quickly declining as the rate of development increases).

### Isolation of *E. coli* from water and sediment

Surface water and sediment samples were collected from natural/open-space locations (S1, M1) to evaluate bacterial contributions from natural or background sources (Fig. 1.) The S1/M1 sampling points are located in the National Forest land. Effluent from three wastewater treatment plants (S11ww, S13 and S14) were also analyzed. All sampling locations and their land use type are listed in Table 1. Water and sediment samples were collected quarterly for 12 months from 20 locations throughout the watershed. All samples obtained from the water surface, and from sediments from the bank of the river were collected in sterile receptacles, stored at 4°C, and analyzed within 6 to 24 hours. For sites that were deep enough to obtain a grab sample, samples were collected about 10–15 cm below the surface of the water. Sites with shallow flow were sampled using a sterile stainless-steel sampling device. All water samples were transported on ice to the laboratory and analyzed by adding 100 ml of water sample to a Colilert vessel and processing following the manufacturer's protocol in accordance with method 9223 [20]. *E coli* populations were expressed as Most Probable Number (MPN/100 ml). For isolation of *E. coli* colonies from Colilert vessels, 100 µl liquid sample was removed from positive wells, then spread plated onto Chromagar ECC agar (CHROMagar Microbiology, Paris, France), and was incubated at 37°C for 24 h.Individual colonies of pure cultures that were isolated were stored at −80°C for further characterization.

**Table 1:** Sampling Locations for MSAR Pathogen Source Evaluation Study.

Site #	Site locations	Land use	Geographic positioning system (GPS)
**S1**	Ice House Canyon	Open Space	N34° 15.057 min.;W117° 37.977 min;1447 m elevation
M1	Cucamonga Creek. @OCWD Ponds	Open Space	San Bernardino County Flood Control District (SBCFCD)
**S2**	Chino Creek @ Central Ave.	Urban runoff	N33° 58.420 min.; W117° 41.302 min;174 m elevation
**S3**	Chino Creek @ Schaefer Ave.	Urban runoff	N34° 00.246 min.; W117° 43.628 min;207 m elevation
**S4**	San Antonio Wash @ County Drive	Urban runoff+Commercial wash out	N30° 01.543 min.; W117° 43.652 min;222 m elevation;
**S5**	Chino Creek. @ Riverside Drive	Urban runoff	N34° 01.144 min.; W117° 44.204 min; 207 m elevation;
**S6**	Cypress Channel @ Schaefer Ave.	Urban runoff only	N34° 00.262 min.; W117° 39.766 min, 208 m elevation;
**S7**	Cypress Channel @ Kimball Ave.	Urban runoff+agricultural	N33° 58.113 min.; W117° 39.624 min, 177 m elevation;
**S8**	Cypress Channel @ Golf Course	Urban runoff	N33° 57.057 min.; W117° 39.555 min;160 m elevation;
**S9**	Big League Dreams storm drain	Urban runoff+possible agricultural runoff during storm events	N33° 57.364 min.; W117° 40.788 min;163 m elevation;
**S10**	Dirt channel on Kimball	Urban Runoff+Agricultural	N33°58.109 min.;W117° 40.286 min 184 m elevation;
S11ww	Cucamonga Creek @ Regional Water Recycling Plant #1	Effluent from wastewater treatment plant	N34°; 01.853 min; W117° 35.946 min; Altitude: 246 m
S11ur	Cucamonga Creek @ Regional Water Recycling Plant #1	Urban runoff+wastewater	N34°; 01.853 min; W117° 35.946 min; Altitude: 246 m
**S12**	Chino Creek @ Pine Ave.	Urban runoff+wastewater	N33°56.941 min.;W117° 39.986 min;155 m elevation;
**S13**	Inland Empire Utilities Agency (IEUA) Regional Water Recycling Plant #5	Effluent from wastewater treatment plant	N33° 57.840 min.; W117° 40.826 min;180 m elevation;
**S14**	IEUA Carbon Canyon Waste Reclamation Facility (CCWRF)	Effluent from wastewater treatment plant	N33° 58.799 min.; W117° 41.655 min;184 m elevation;
ST2	Santa Ana River @ Prado Dam	Urban Runoff+Agr	N33°; 54.737 min; W117° 38.711 min Altitude: 141 m.
C3	Prado Park outlet	Urban Runoff+waste water discharge	N33°; 56.402 min; W117° 38.763 min Altitude:166 m
ST5	Santa Ana River @ River road	Urban Runoff+Agr	N33°; 55.405 min; W117° 35.894 min Altitude:155 m.
M5	OCWD (Prado)Wetlands Effluent	Wetland treated (bacteria loaded) Orange County Water District (OCWD	N33°; 54.737 min; W117° 38.711 min Altitude: 141 m

Sediment samples from the 0- to 10-cm depth were taken from the creek or river banks using ethanol-disinfected core tubes and stored in Whirl-Pak bags at 4°C until processed; usually within 24 h. Moist sediment samples (10 g) were diluted with 90 ml of tryptic soy broth (TSB) and shaken for 15 minutes. Ten ml of the suspension was added to a Colilert vessel, diluted 1∶10 and mixed. One ml from the 1∶10 dilution was transferred to another vessel and was further diluted 1∶1,000, and an aliquot was added to the Colilert media, mixed, then sealed in QuantiTrays and incubated at 37°C for 24 h. Cultures purified on plate count agar plates were suspended in sterile 50% (vol/vol) glycerol, and were transferred to cryovials and 96-well cell culture plates, and were stored at −80°C until they were used for analyses. Isolates were confirmed as *E. coli* using API20E strips (bioMérieux, Paris, France), and were genetically confirmed using the *uidA* primer pair [21]. *E. coli* strain ATCC 25922 and *Klebsiella pneumoniae* ATCC 35657 served as positive and negative controls, respectively, for all tests. Four confirmed isolates and when possible up to six isolates from each site were stored giving a total of about 2,000 *E. coli* isolates in our collection. Out of these, 600 representative isolates were used for PFGE and antimicrobial activities. The 600 isolates were selected based on the percentage of isolates collected from each source and stored in our collection.

### Genetic diversity of *E. coli* isolates using pulsed-field gel electrophoresis (PFGE)

PFGE was conducted to assess the genetic diversity of *E. coli* isolates to analyze genetic similarities between surface and sediment samples as well as among the different sources or sites. Isolates were subtyped based on PFGE patterns of *Xba*I-digested genomic DNA fragments in accordance with the standard protocol established by the Centers for Disease Control and Prevention (PulseNet; Centers for Disease Control and Prevention, Atlanta, GA). Bacterial strains were grown overnight on tryptic soy agar (TSA) plates at 37°C. Bacterial colonies were suspended in cell suspension buffer and adjusted to an optical density (OD) of 1.3–1.4 using a spectrophotometer set at 590 nm. The 400 µl adjusted cell suspension was mixed with 20 µl of proteinase K and an equal volume (400 µl) of melted 1% SeaKem Gold Agarose (BioWhittaker, Rockland, ME, USA) containing 1% sodium dodecyl sulfate. The mixture was carefully dispensed into appropriate wells of a reusable plug mould (Bio-Rad Laboratories, Hercules, CA). After solidification, the plugs were transferred individually to round bottom tubes containing 1.5 mL of cell lysis buffer (50 mmol l^−1^ Tris–HCl, 50 mmol l^−1^ EDTA, pH 8·0; 1% sarcosine) and 0.5 mg ml^−1^ of proteinase K. Cells were lysed in a 54°C water bath for 2 h with constant and vigorous agitation at 175–200 rev min^−1^. After lysis, the plugs were washed twice with preheated water and four times with preheated TE buffer for 10–15 min per wash at 50°C, with agitation as above. Plugs were then stored in 2 ml of TE buffer at 4°C until they were ready for DNA restriction enzyme (RE) digestion. The DNA in agarose plugs was digested with 50 U of *Xba*I for at least 3 h at 37°C in a water bath. The plugs were loaded onto the wells in a 1% (wt/vol) pulse-field-certified agarose gel. DNA restriction fragments were separated using a CHEF-MAPPER (Bio-Rad Laboratories) with pulse times of 5–50 s at 14°C for 14 h in 0.5× TBE buffer at 6 V cm^−1^. The gel was stained with ethidium bromide (Sigma-Aldrich, St. Louis, MO), and restriction fragment patterns were photographed using a Gel Documentation system (Bio-Rad). *XbaI*-digested *Salmonella enterica* serovar Branderup H9812 was used as a molecular weight marker and included in the first, middle, and last lanes of all gels to account for run-to-run variability. Comparison of digested profiles to identify restriction enzyme digestion pattern clusters (REPCs) was performed with the BioNumerics software, version 5.0 (Applied Maths, Austin, TX). Fingerprints were clustered by using the Jaccard coefficient evaluated by the unweighted-pair group method (UPGMA). A tolerance and optimization of 1% was allowed to account for gel-to-gel differences. Isolates that had ≥90% pattern similarity were considered highly closely related and were grouped as a cluster or clonal population. Patterns that did not fall into any particular REPC were also assigned a pattern identification number, and if they were detected only once during the trial they were considered unique. Isolates were considered indistinguishable if they had the same number and size of bands in a PFGE fingerprint pattern. Isolates were considered to be closely related if their PFGE pattern differed by changes consistent with a single genetic event. Such strains typically do not have differences in more than three bands.

### Antimicrobial susceptibility testing

Antimicrobial susceptibility tests (phenotypes) of ∼600 generic *E. coli* isolates from sediment and surface water were performed using a disk diffusion assay following CLSI standards [22]. Mueller-Hinton II agar (Difco) was used and cells were harvested from the surface of the medium with a cotton swab after 24 h growth at 37°C. Cells were suspended in sterile saline (0.85% NaCl) and cell density was adjusted to a 0.5 McFarland turbidity standard. Diluted cells were spread plated onto the surface of agar plates, and antibiotic disks were placed on the surface. Following incubation (24 h at 37°C), zone sizes (diameter) were measured (mm) to two decimal points and were used for quantitative analysis. Isolates resistant to two or more antimicrobials were defined as multiple drug resistant. *E. coli* ATCC 25922 (American Type Culture Collection, Manassas, VA) was included in each assay as a negative control strain. Antimicrobial agents were tested with BD BBL Sensi-Disc antimicrobial susceptibility test discs (Becton Dickinson & Co., Sparks, MD) using the following breakpoints (µg: Amoxicillin/clavulanic acid - 20/10 µg, ampicillin - 10 µg, cephalothin - 30 µg, erythromycin - 15 µg, rifampin - 5 µg, streptomycin - 10 µg, and tetracycline - 30 µg.

### Antimicrobial resistance gene detection

Antimicrobial resistance genes were analyzed in 53 *E. coli* isolates that were not identified as belonging to any of the clonal populations. All 53 isolates were treated as unique isolates and antimicrobial susceptibility tests and PFGE analysis were conducted on these samples for the second time. As before *E. coli* 25922 was used as a negative control. Multiplex PCR screens (Table 2) were performed on the 53 unique *E. coli* samples using *Int* I/*Sul* I and *Int* 2/*dhfr*I primer pairs. Genes encoding for ampicillin resistance (*bla*_TEM_), tetracycline resistance (*tet* A, *tet* B, and *tet* C), and streptomycin resistance (*aadA*I) were also characterized. Details of primers, annealing temperatures, and amplicon sizes are provided in Table 2 [46]–[50].

**Table 2:** Antimicrobial families, genetic markers, and primer sequences, for resistance genes tested.

Antimicrobial Family	Genetic marker	Primer name	PCR primer sequence (5′-3′)	AnnealingTemp °C	Amplicon size (bp)	Source primer sequences
**Beta-lactams**	*bla*_TEM_	Bla F	GAGTATTCAACATTTTCGT	50	857	48
		Bla R	ACCAATGCTTAATCAGTGA	50		
**Aminoglycosides**	*ant*(*3*″)*-Ia* (*aadAI*)	aadA F	CATCATGAGGGAAGCGGTG	50	786	46
		aadA R	GACTACCTTGGTGATCTCG	50		
**Tetracycline**	*tet*(A)	tetA F	GTGAAACCCAACATACCCC	50	888	46
		tetA R	GAAGGCAAGCAGGATGTAG	50		
	*tet*(B)	tetB F	CCTTATCATGCCAGTCTTGC	50	774	46
		tetB R	ACTGCCGTTTTTTCGCC	50		
	*tet*(C)	tetC F	ACTTGGAGCCACTATCGAC	50	881	46
		tetC R	CTACAATCCATGCCAACCC	50		
**Trimethoprim**	*dhfrI*	dhfrI F	AAGAATGGAGTTATCGGGAATG	50	391	46
		dhfrI R	GGGTAAAAACTGGCCTAAAATTG	50		
	dfrA1-like	dfr-F	CCCAACCGAAAGTATGCGGTCG	48	171	50
		dfr-R	GTATCTACTTGATCGATCAGG	48		
**Class 1 integron**	intI1	int-F	GCCACTGCGCCGTTACCACC	60	898	47
		int-R	GGCCGAGCAGATCCTGCACG	60		
**Class 2 integron**	intI2	intI2F	GCAAATGAAGTGCAACGC	48	466	49
		intI2R	ACACGCTTGCTAACGATG	48		
**Sulfonamides**	sul1	sul1-F	CGGCGTGGGCTACCTGAACG	60	433	47
		sul1-R	GCCGATCGCGTGAAGTTCCG	60		

### Statistical analysis

*E. coli* CFU counts were averaged from replicate plates. The counts were log transformed, and the mean and variance were calculated for each site before performing the analyses of variance (ANOVA) [23]. Cluster analysis was performed on PFGE clonal types by Jaccard comparison using the UPGMA method.

## Results

### *E. coli* recovery from sediment and surface water

**Figure 2:**
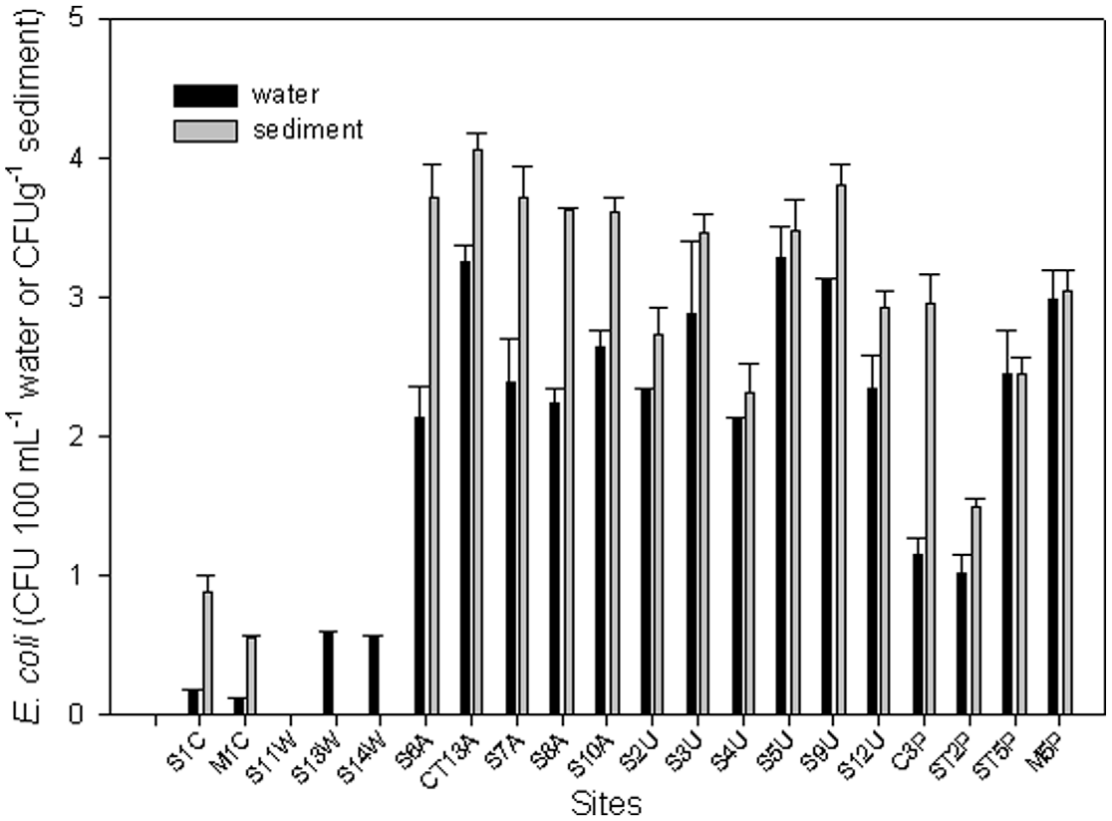
The abundance of *E. coli* isolates in sediment and surface water samples (n = 450) collected from 20 sites over a 12-month period. Counts ranged from undetectable (detection limit 1 CFU 100 ml^−1^), in the surface water, to 2.5×10^4^ CFU 100 ml^−1^ in the sediment. Sample names on the X axis are as shown in Figure 1 and Table 1 with letters C, W, A, U and P representing control sites, WWTPs, agricultural runoff, urban runoff and Prado Park recreational area. Samples are S1C and M1C (control sites); S11W, S13W, and S14W were collected from the outlets at the three WWTPs; S6–S10A from agricultural inputs; S2U to S12U are from urban runoff, C3P, ST2P, ST5P, and M5P are from locations in and around the Prado recreational park. Error bars represent standard errors of two replicate samples.

The abundance of *E. coli* in sediment and surface water was determined on 450 water and sediment samples collected from 20 sites over a 12-month period. Counts ranged from undetectable (detection limit 1 CFU 100 ml^−1^) in the surface water to 2.5×10^4^ CFU 100 ml^−1^ in the sediment (Fig. 2). A total of 60 water samples (12.5%) had *E. coli* counts at or below the EPA Water Quality Objectives of 120 CFU 100 ml^−I^. These samples were collected from the control sites (SI and MI) at about 1447 m elevation in the St Gabriel Mountain and the other samples (S11WW, S13W, and S14W) were collected from the outlets at the three waste water treatment plants (WWTPs). The 14 remaining sites differed consistently in degree of contamination with sites S6–S10A (agricultural impact) and sites S2U to S12U (urban runoff) with higher *E. coli* concentrations on average than site ST2P, which is located about 1 km from Prado Dam (Fig. 1). This is the final effluence water from Prado Park/wetland/dam. Concentrations of *E. coli* were significantly lower (*P* = 0.05) at this site compared to the other sites that were impacted by agricultural activities and urban runoff, and site ST5, which is the influent water to Prado Park/wetland/dam (Fig. 2). Comparison of *E. coli* concentrations of sediment and surface water using the t-test [23] showed that sediment concentrations were significantly (*P* = 0.0012) higher than surface water samples (Fig. 2).

### Diversity of *E. coli* isolates using PFGE

The change over time and sources (sites) in *E. coli* diversity in surface water and sediment was monitored throughout the watershed during the study. PFGE was performed on 600 *E. coli* isolates to determine their diversity (Table 3). Using Jaccard similarity coefficients and UPGMA, strains with PFGE fingerprint patterns with ≥90% similarity were considered clonal populations. A summary of *E. coli* PFGE restriction pattern diversity showed that 465 isolates were grouped into six clonal populations. Each cluster had clonal populations ranging from 10 to 137 isolates (Table 3). Fifty three additional isolates were treated as unique isolates because they did not cluster within the six clonal populations. Data analysis was conducted based on sources of *E. coli* such as agricultural input from the Cypress channel (S6, S7, and S8), urban runoff from Chino Creek (S2, S3, S4, S5, S9, and S12), WWTPs (S11WW, S13 and S14), control sites at about 1,447 m elevation (S1, M1), and from the Prado park area which is used for non-contact recreational activities (C5, M5, ST2, and ST5). The most diverse isolates were collected from sediment and surface water in the Chino Creek, which is dominated by inputs from urban runoff or human activities (Table 3; Fig. S1 a and b). In the sediment, five out of six clonal populations were determined in December and June compared to only three in April (Fig. S1 a). In the surface water, all six clonal populations were observed in December and in June, compared to five in April (Fig. S1b). In the Cypress channel that received mainly agricultural inputs, *E. coli* isolates were less diverse than those from Chino Creek. Higher diversity was found in samples collected in December than in April and June samples (Fig. S1c). Very few samples were collected from sediment for comparisons because S6 is lined with concrete. Two WWTPs were sampled in April and June but sampling was not conducted in December due to lack of access. As was shown from the previous two channels, *E. coli* isolates associated with the WWTPs were more diverse in June than in April (Fig. S1d). The pattern of diversity of *E. coli* isolates in the Prado Park area was very similar to that of the Chino Creek, with higher diversity obtained in the water compared to sediment samples. In the sediment samples (Fig. S1 e) four out of six clonal populations were observed in December and June compared to only three clonal populations in April. In the surface water, five clonal populations were observed in December, April, and June (Fig. S1f). About 32% and 47% of isolates in the surface water were more diverse than isolates in sediment from Chino creek and Cypress channel, respectively. However, diversity of isolates was about the same in both surface water and sediment in both Prado park sites and the control sites (Table 3).

**Table 3:** Genotypic diversity of *E. coli* isolates from the Middle Santa Ana River watershed derived from major sources by PFGE.*

cluster series	# of clusters	Total isolates	Sediment isolates(Chino Creek)	Sediment isolates(Cypress channel)	Water isolates(Chino Creek)	Water isolates(Cypress channel)	Sediment isolates(Prado)	Sediment isolates(WWTP)	Water isolates(Prado)	Water isolates(WWWTP)	Sediment isolates(Cont.)**	Water(Cont.)
1	137	137	21	9	43	18	12	2	17	11	1	3
2	62	124	21	10	25	20	12	0	16	12	4	4
3	28	84	12	6	12	6	18	6	12	9	3	0
4	14	56	8	4	8	4	13	4	7	4	0	4
5	2	10	0	0	5	0	0	0	0	0	0	5
6	9	54	6	0	6	6	6	6	12	6	6	0
Total	**252**	**465**	**68**	**29**	**99**	**54**	**61**	**18**	**64**	**42**	**14**	**16**

*Isolates demonstrating PFGE patterns with ≥90% similarity were considered clusters using Jaccard similarity coefficients and UPGMA analysis.

**Cont.; control site.

### Prevalence of *E. coli* isolates with antibiotic resistance phenotypes

**Figure 3:**
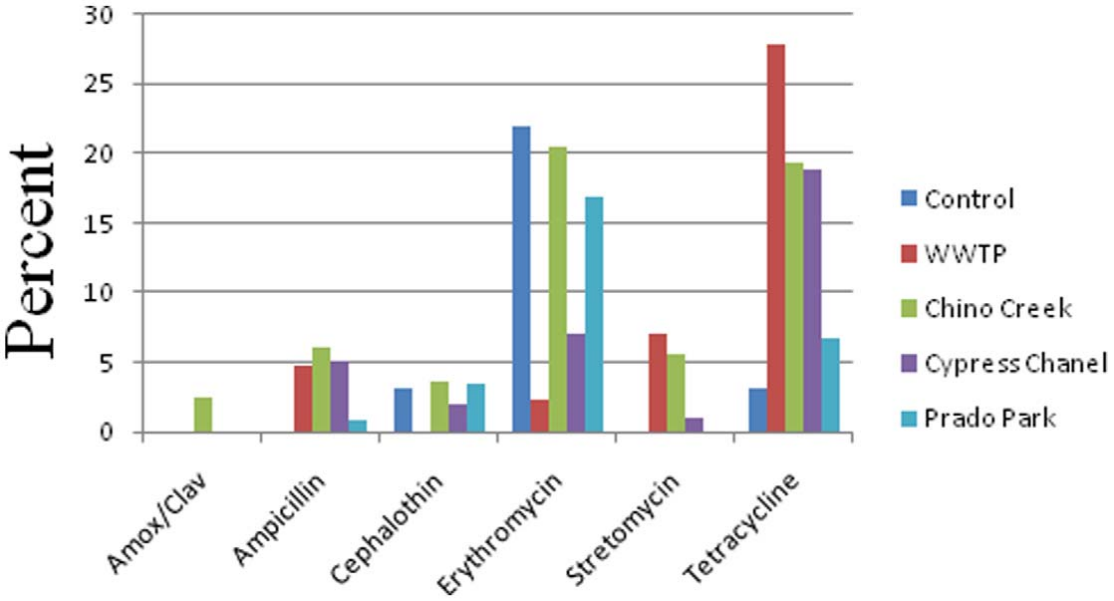
Antimicrobial resistance (%) from five zones within the watershed. A total of 600 isolates were characterized for antimicrobial sensitivities.

Eight antibiotics were used for susceptibility tests of *E. coli* isolates. Resistant phenotypes were determined for 600 isolates from the control, WWTPs, Chino Creek, Cypress channel, and Prado Park area (Fig. 3). A total of 88–95% of *E. coli* isolates were resistant to rifampicin; these data are therefore not presented in Figure 3. The second most prevalent antibiotic resistance was demonstrated against tetracycline. The highest resistance to tetracycline was found in samples collected from WWTPs. This was followed by isolates associated with urban runoff and agricultural activities. The third most prevalent resistance was demonstrated against erythromycin. Most *E. coli* isolates with resistance to erythromycin were found in the control sites, Chino creek, and Prado park area. Resistance to the remaining antimicrobials was minimal (<7%), and amoxicillin resistance was only detected in isolates from urban runoff from Chino creek. Site by site analysis (data not shown) showed that rifampicin resistance was present in 93% (n = 75) of isolates from the control and WWTPs, 94.4% (n = 178) of isolates from Chino creek, 97.2% (n = 107) of isolates from Cypress channel, and 97.5% (n = 119) of isolates from Prado park area.

### Characterization of *E. coli* isolates with multiple antimicrobial resistance (AMR) profiles

Twenty four percent (144 isolates) of the 600 isolates were resistant to more than one antimicrobial (Table 4). The antimicrobials associated with most multiple resistances were rifampicin, tetracycline, and erythromycin. Four percent (n = 24) of the isolates from the Chino creek sediments were resistant to ≥2 antimicrobials. A total of 7.5% (45 isolates) in the Chino creek surface water, 4.5% (27 isolates) in the Cypress channel in both sediment and surface water, 1.3% (8 isolates) in control sites, 2.2% (13 isolates) in WWTPs, and 4.5% (27 isolates) from the Prado park sediment and surface water were resistant to ≥2 antimicrobials. Five isolates from site S2 (sediment; Fig. S2a) along Chino creek had multiple resistances to seven antimicrobials, making isolates from this site the most resistant group. This was followed by isolates from a sediment sample from site S12 (Chino creek), a sample from urban runoff, then site M5 in the Prado Park, which is a sediment sample. Isolates with most multiple AMR were found in sediment and mainly from inputs associated with urban runoff along Chino creek (Table 4).

**Table 4:** Multiple antimicrobial resistant *E. coli* isolates from the major sources.

Antimicrobial	Total isolates 144	Sediment isolates 24(Chino Creek)	Sediment isolates 10(Cypress channel)	Water isolates 45(Chino Creek)	Water isolates 17(Cypress channel)	Sediment isolates 11(Prado)	Water isolates 16(Prado)	Water isolates 13(WWTP)	Sediment isolates 5(Cont.)*	Water 3(Cont.)
Tetracycline	**48.6 (70)**	**37.5 (9)**	**60 (6)**	**37.7 (17)**	**74.4 (13)**	**45.5 (11)**	**18.7 (3)**	**100 (13)**	**20.0 (1)**	**100 (3)**
Streptomycin	**11.1 (16)**	**4.1 (1)**	**0 (0)**	**24.4 (11)**	**5.8 (1)**	**0 (0)**	**0 (0)**	**23.0 (3)**	**0 (0)**	**0 (0)**
Rifampicin	**96.5 (139)**	**100 (24)**	**100 (10)**	**95.5 (43)**	**100 (17)**	**100 (11)**	**100 (16)**	**100 (13)**	**100 (5)**	**0 (0)**
Erythromycin	**50.6 (73)**	**50 (12)**	**30 (3)**	**60.0 (27)**	**36.3 (4)**	**36.3 (4)**	**93.7 (15)**	**7.6 (1)**	**80 (4)**	**100 (3)**
Cephalothin	**8.3 (12)**	**16.6 (4)**	**0 (0)**	**6-6 (3)**	**11.7 (2)**	**9.1 (1)**	**6.2 (1)**	**0 (0)**	**0 (0)**	**33.3 (1)**
Ampicillin	**12.5 (18)**	**8.3 (2)**	**30(3)**	**17.7 (8)**	**11.7 (2)**	**9.1 (1)**	**0 (0)**	**15.3 (2)**	**0 (0)**	**0 (0)**
Amox/Clav	4.8 (7)	16.4 (4)	0 (0)	2.2 (1)	0 (0)	9.1 (1)	6.2 (1)	0 (0)	0 (0)	0 (0)

*Cont.; control site.

Detailed examination of each channel, such as the Chino creek sediments, showed that sites S11ur and S9 sediment samples had one isolate each with resistance to two antimicrobials (Fig. S2a). For site S12 (sediment), as an example, four out of five isolates were resistant to tetracycline and an additional antimicrobial. All five isolates were resistant to rifampicin and at least one other antibiotic. The resistance pattern of isolates from surface water in the Chino creek was more complex than sediment samples since more isolates (45) expressed multiple AMR in surface water (Table 4 Fig. S2b) than the 24 isolates from sediment samples. In the Cypress channel, with input from agricultural activities (CAFOs), more isolates (17) expressed multiple AMR in surface water than in sediment (Fig. S2 c). Isolates from sediments may be resident populations, whereas those in water samples are transient populations transported from upstream of the channel to downstream depending on water volume and flow velocity. In the control sites (Fig. S2 d), one isolate from surface water expressed multiple AMR to rifampicin, erythromycin, and cephalothin, while two isolates from sediment expressed dual AMR to rifampicin and erythromycin. These samples were isolated from an elevation of about 1447 m. Samples from the M1 control site located on the foothill of the mountain showed more isolates expressing multiple AMR phenotypes mainly to rifampicin, erythromycin, and tetracycline. From the three WWTPs, 13 isolates expressed multiple AMR phenotypes with resistances to tetracycline and rifampicin (Table 4, Fig. S2e). In the Prado park area (Table 4, Fig. S2f), the resistance pattern followed what we observed in the Cypress channel with more isolates from water samples (17) expressing multiple AMR than from sediment (10).

### Prevalence of antimicrobial resistance (AMR) genes in *E. coli* isolates

**Figure 4:**
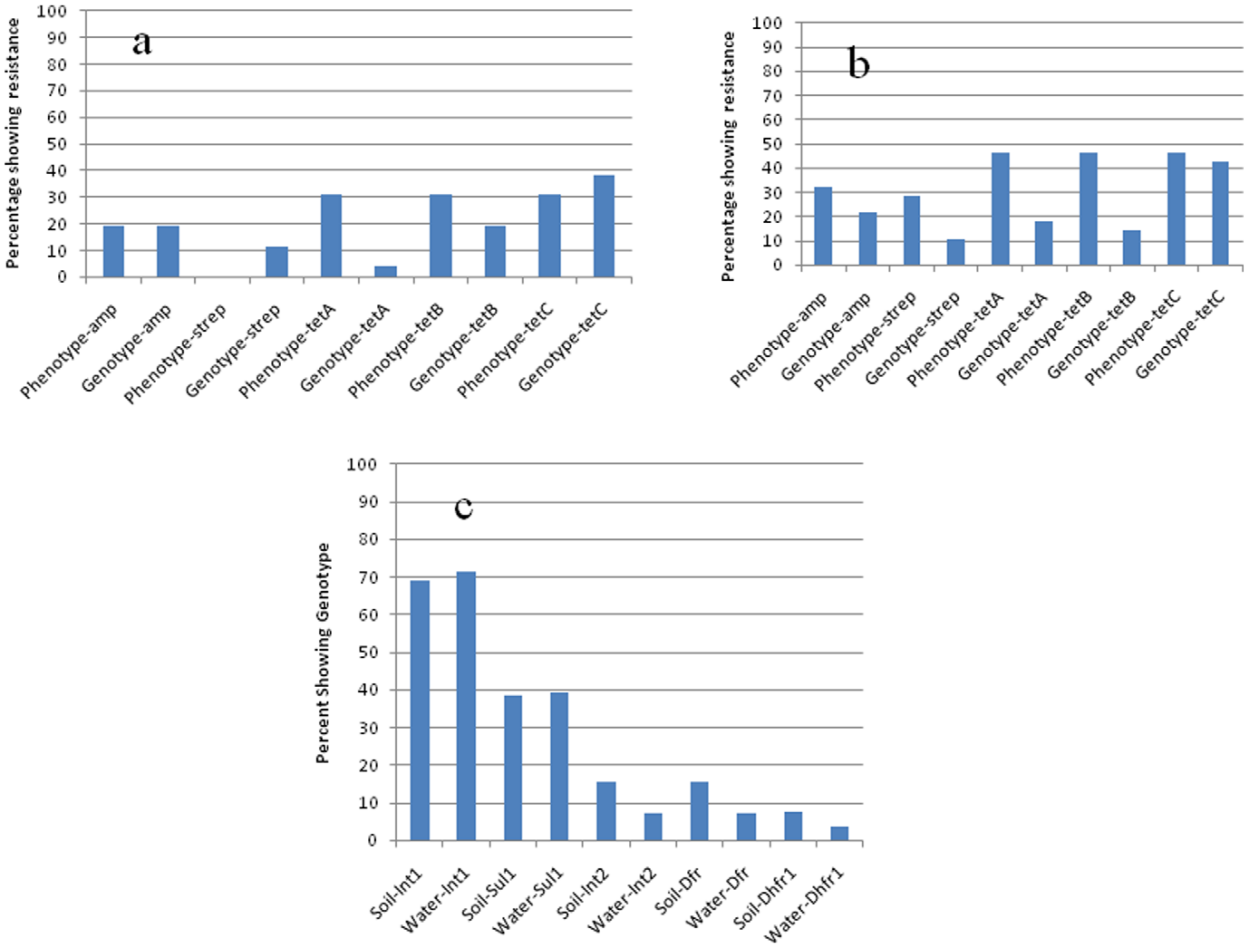
Correlation of antimicrobial susceptibility and presence of resistance gene sequences of *E. coli* isolates from (a) sediment and (b) surface water. Integrons in sediment and surface water (c).

**Figure 5:**
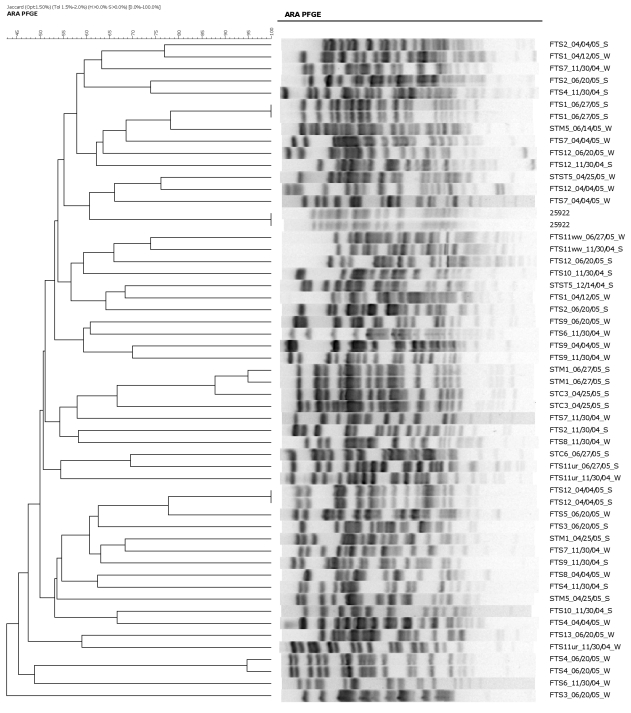
Representative PFGE fragment patterns and dendrogram analysis of unique *E. coli* isolates obtained from surface water and sediment showing diversity and stability of isolates.

Antimicrobial resistance genes were analyzed in 53 *E. coli* isolates that did not fall into clonal populations identified by PFGE in Table 3. All 53 isolates were treated as unique isolates and antimicrobial susceptibility tests and PFGE analyses were performed on these samples for the second time (Fig. 4, Fig. 5). Genes for ampicillin resistance (*bla*_TEM_) and streptomycin resistance (*ant3*″)*-Ia* (also called *aadA1*) were detected at lower frequencies than *tet* genes (Fig. 4a and b). Markers for integrons were detected in approximately 70% (n = 38 s) of the 53 isolates studied. Class I integrons were detected in 37 (69%) isolates from sediment and from 38 (71%) of 53 isolates from surface water, while class II integrons were detected in 8 (15%) isolates from sediment and 4 (7%) isolates from surface water (Fig. 4c).

When the 53 unique isolates were reanalyzed for antimicrobial susceptibility and presence of resistance genes in *E. coli* isolates, genotypes did not always correspond with the phenotypic expression of individual isolates (Fig. 4). Ten isolates (19%) from sediment samples were resistant to ampicillin and they also carried the *bla*_TEM_ gene (Fig. 4a). These were the only samples that showed 100% agreement of phenotypic resistance and presence of genes encoding the phenotype. Six isolates (11%) carried the gene for streptomycin resistance, but none expressed the resistance phenotype. Sixteen isolates (30%) expressed phenotypic resistance to tetracycline, but resistance genes were found in two isolates (4%) for *tet*A, 10 isolates (19%) for *tet*B, and 20 isolates (38%) for *tet*C from the 53 unique isolates (Fig. 4a). There were 15 isolates (29%) that were resistant based on susceptibility to streptomycin, but only 6 isolates (11%) had the *aadA*I gene marker (Fig. 4b). Twenty isolates (46%) expressed phenotypic resistance to TetA, TetB, and TetC; of these isolates, 9 (18%) had *tet*A, 8 (14%) had *tet*B, and 23 (43%) had *tet*C (Fig. 4b). Sulfamethoxazole-resistance was demonstrated by 20 (38%) of isolates from sediment and 21 (39%) from surface water. Trimethoprim-sulfamethoxazole resistance was associated with presence of the *dhfrI* gene sequence and was found in two isolates from surface water and eight isolates from sediment.

A majority of the 53 *E. coli* isolates showed unrelated PFGE patterns except for the control strain *E. coli* 25922 and four other isolates from sites FTS4-06/20/05-W, FT12-04/04/05, STM106/27/05, and FTS1-06/27/05 (Fig. 5). These four isolates and the control were run in duplicate to evaluate the genetic stability of some *E. coli* isolates. The repeatable PFGE patterns confirmed that the 53 isolates were truly unique and their genetic profiles were very different.

## Discussion

### Prevalence of *E. coli* in the watershed

In this study, the concentration of *E. coli* in the two major creeks and the Prado area exceeded the single sample objectives for *E. coli*: 235 CFU/100 mL, according to the United States Environmental Protection Agency (USEPA) that is used in many parts of the country for National Pollutant Discharge Elimination System and Total Maximum Daily Load purposes [24]. Based on high numbers of *E. coli*, the MSAR was included in the lists of bodies of water with bacterial counts above the 1995 limits. The elevated *E. coli* counts along the Chino Creek and the Cypress channel coincide with mostly nonpoint sources of fecal contamination. All sampling sites along the Chino Creek and Cypress channel were in violation of local and EPA water quality standards for fecal indicator bacterial counts. All of these sites are situated near known human point sources or agricultural operations [25], [26], [27]. Due to high concentrations of indicator bacteria in the MSAR, some remediation activities had been instituted to reduce some of the contaminants. About 50% of water from the Santa Ana River flows through 50 ha of surface flow constructed wetland (Prado wetland) and the Prado Dam. Samples for inflow into the wetland were collected at site ST5 and outflow at site ST2. As seen in Fig. 2, the concentrations of *E. coli* from site ST2 after flowing through the wetland and the dam were significantly reduced compared to the concentrations from site ST5. This agrees with our previous study which showed that the wetland may be a very good system for the removal of contaminants from surface water [26]. Another reason may be the continuous exposure of the water to ultra violet light at Prado dam. Half of the water from Santa Ana River is used for ground water recharge to protect the aquifer against salt water intrusion from the Pacific Ocean and the remaining 50% empties into the Pacific Ocean. For bodies of water used for non-contact recreational and contact recreational purposes, high densities of bacteria can result in immediate closure for public use. For example, concentrations of *E. coli* along Cypress channel and Chino Creek were strongly influenced by land use. The high densities of *E. coli* in this watershed are subjected to different environmental pressures that likely result in genetically diverse populations.

### Diversity of *E. coli* isolates in the watershed

This study showed that *E. coli* isolates were more diverse in surface water than in sediment using PFGE fingerprinting, and also suggests that more *E. coli* isolates in the sediment were resident populations. Our PFGE data showed considerable genetic diversity among *E. coli* isolates from water and sediment samples from the same location collected at the same time over the experimental period from all the sampling sites throughout the watershed. Multiple isolates obtained from the same sampling location, the same sample type and sampling period showed genetic diversity. In the sediment, *E. coil* isolates were more stable. Consequently, the sediment isolates that we examined appeared to be reservoirs for *E. coli*, whose presence over time may not solely be due to the emergence of new genotypes into the sediment from local or transient point and nonpoint sources, but a function of survival and proliferation of some residence populations [28]. This suggests the presence and growth of naturalized *E. coli* populations in sediment samples [2]. Alternatively, it has been suggested that genomic rearrangement during the survival and persistence of these enteric bacteria is a possibility [29], [30], [31].

These results showed that there is a significantly higher *E. coli* diversity in water than associated sediment. The higher diversity of isolates from surface water in Prado area agrees with our finding of higher diversity in surface water in Chino creek, again suggesting that *E. coli* populations in surface water were more diverse than the population in the sediment, and indicates that more *E. coli* isolates in the sediment were resident populations. Additionally, the PFGE patterns of *E. coli* in sediment and water raised the question regarding the applicability of sensitive fingerprinting techniques such as PFGE for microbial source tracking. PFGE has been standardized and used extensively, and will continue to be the standard for discerning genetic relatedness among isolates. The results of this study show that even a minor change in the banding pattern can significantly affect clusters. Thus, to employ PFGE for source tracking in a large watershed like the Santa Ana River, a very extensive PFGE fingerprint library is needed. The DNA fingerprint library has to be comprehensive enough to account for the potential multiple contaminant sources and accommodate the spatial and temporal genetic variability of *E. coli* strains. The library should also take into account the possible genetic diversity fluctuations that can occur within strains during survival in such environments.

### Prevalence of multiple antimicrobial resistances (AMR) in *E. coli* isolates

Most *E. coli* isolates were resistant to ampicillin, erythromycin, streptomycin, tetracycline, and cephalothin (Fig. 3). The resistance by all eight isolates from the control sites to rifampicin and erythromycin may suggest that these two antimicrobials are naturally present in the mountain region of the study sites or naturally present throughout the watershed since they were the most common antimicrobial resistance detected in our samples. It is noteworthy that, although ampicillin and tetracycline are old antimicrobials, they are still widely used. The relatively high rates of tetracycline resistance among *E. coli* isolates from WWTPs were unexpected considering tetracycline is used less frequently in humans than in animals (Fig. 3). About 72% of our unique isolates (38 out of 53 isolates) from sediment and surface water possessed a class 1 integron. Four different integron classes have been characterized, with class 1 being the most common among clinical isolates of *E. coli* [32], [33]. Resistance genes are often associated with integrons or mobile DNA elements such as plasmids and transposons that facilitate the spread of resistance genes [32], [33], [34], [35]. More often, there is a linkage between many of these resistance genes on mobile elements and the distribution of antibiotic resistant bacteria in the environment [35], [36], [37]. While we did not study the exact mechanisms of resistance in the current work, previous molecular studies have shown strong statistical associations between different resistance genes in *E. coli* isolates [37], [38], [39]. In this study, we found a strong association between certain phenotypes and genotypes, indicating that the resistance to a given antimicrobial was likely caused in some cases by a single gene. In some instances, the antimicrobial resistance phenotype and genotype did not correlate. For instance, we detected both 19.2% phenotype and genotype with the *bla*_TEM_ gene for ampicillin, and 15.5% *aadA*I gene for streptomycin, but no phenotypic resistance to streptomycin (Fig. 4a) in the isolates from sediment, whereas in the isolates from surface water, genotypic and phenotypic expression were quite different. Antibiotic resistant phenotypes can emerge from many different genetic determinants, and each determinant may present unique epidemiological features [35], [40].

Tetracycline resistance was by far the most common type of resistance observed in *E. coli* isolates associated with WWTPs and this was linked to human origin (Fig. 3). No differences in resistance to this antibiotic were found in isolates from urban runoff samples and from isolates from the Cypress channel. This is not surprising as the Cypress channel receives input from CAFOs where tetracycline is often used as a first-line antimicrobial in disease prevention and growth promotion in food animals [41], [42], [43]. Tetracycline resistance genes are located on mobile genetic elements, and can be transmissible between bacteria [44]. Resistance to amoxicillin-clavulanic acid in *E. coli* isolates was mainly found along urban runoff samples collected along Chino creek. This is not surprising since the use of this antimicrobial is related to different therapeutic end use for human diseases. The correlation between antimicrobial resistance and the presence of multiple AMR genes was very high in some instances. For streptomycin, a discrepancy between genotype and phenotype among isolates from sediment was expected, because previous studies have shown that streptomycin resistance genes can be detected in isolates classified as susceptible, suggesting that the breakpoint used for this antimicrobial may be too high for epidemiological purposes [38], [40], [45]. However, in isolates from surface water, the frequency of the resistance phenotype was higher than presence of related AMR genes. This appears to suggest that other resistance phenomena could be at play.

PFGE analysis showed that *E. coli* isolates were very diverse and there was no evidence that a small number of environmentally-adapted isolates represented a dominant population from surface water or sediment throughout the watershed. Therefore, to employ PFGE for source tracking in a large watershed like the Santa Ana River watershed, a very extensive PFGE fingerprint library is needed. Key steps are needed for the full understanding of the level of selection pressure that is imposed on selected bacterial populations in such an environment; the DNA fingerprint library has to be comprehensive enough to account for the potential of multiple contamination sources and to accommodate the spatial and temporal genetic variability of *E. coli* strains. The library should also consider the possible genetic diversity fluctuations that can occur within strains during survival and growth in such environments. The use of a variety of personal care products in household settings, e.g., pharmaceutical compounds such as antibiotics for different therapeutic and subtherapeutic end points in urban environments may contribute more substantially to emergence of antimicrobial resistance in the environment than previously thought. Antibiotic use selects for antibiotic resistance regardless of why it is use, and this threatens public health when important evolutionary events occur first in bacterial populations in the environment and then move into the bacterial populations associated with humans.

## Supporting Information

Figure S1 **The pattern of diversity of** ***E. coli*** **isolates in the MSAR watershed: (a) soil from Chino creek, (b) water from Chino Creek, (c) water from Cypress channel, (d) water from WWTPs, (e) soil from Prado, and (f) water from the Prado park area.** Sample names on the X axis are as shown in Figure 1 and Table 1 followed by dates that samples were collected from the different sites.
(PDF)

Figure S2 **Multiply antimicrobial resistant** ***E. coli*** **isolates.** The bars in Figure 5a as an example shows; S12-s, S2-s, and S3-s showed each site with five, five, and nine isolates, respectively, with multiple antimicrobial resistance phenotypes. S after the site names in Table 1 indicates the sample was taken from sediment.
(PDF)
