# Supplementary figures and images for: Genetic Diversity and Antimicrobial Resistance of Escherichia coli from Human and Animal Sources Uncovers Multiple Resistances from Human Sources
Source: PLoS One. 2011 Jun 8;6(6):e20819. doi: 10.1371/journal.pone.0020819 (PMC3110821; doi:10.1371/journal.pone.0020819)

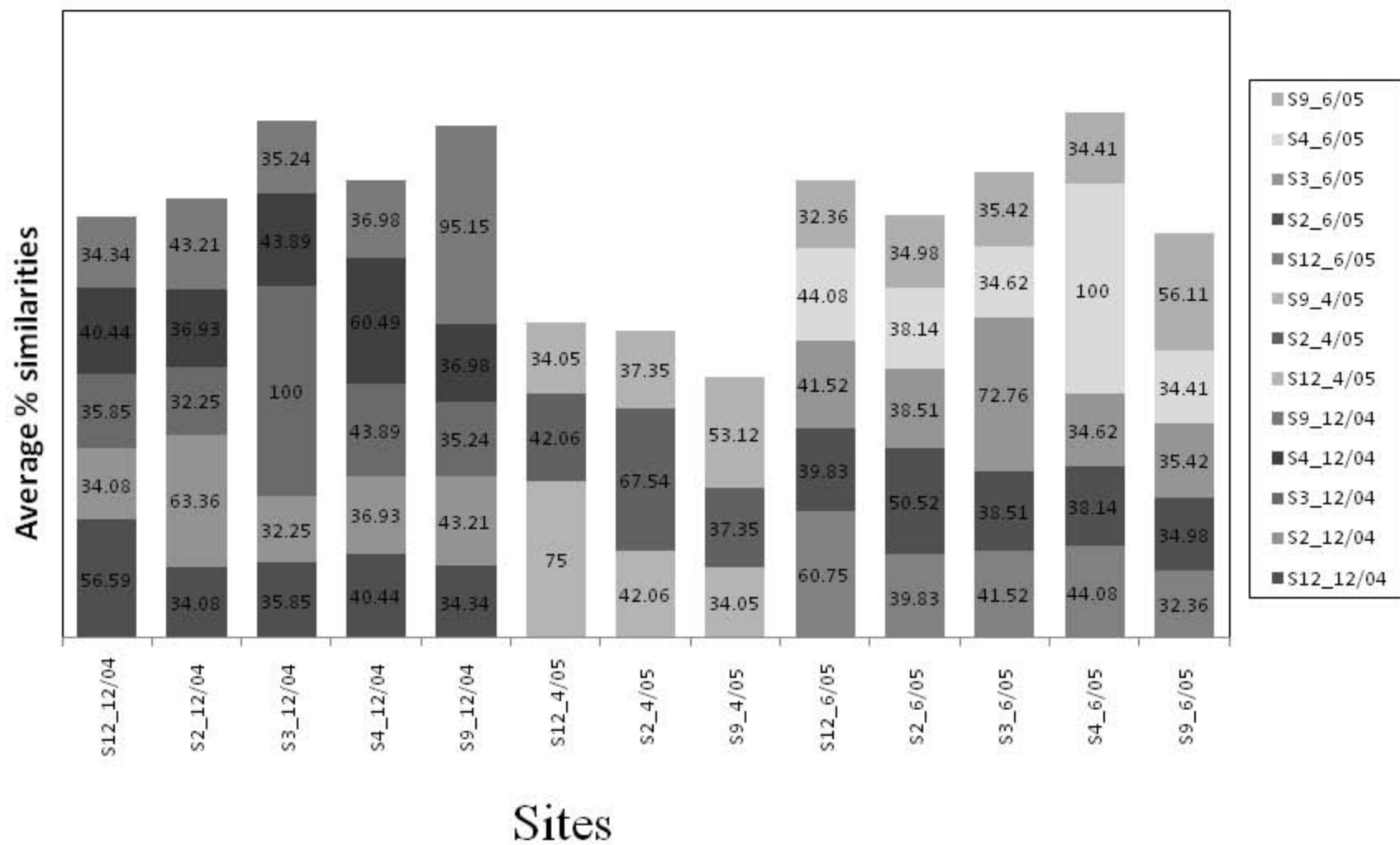

Average % similarities

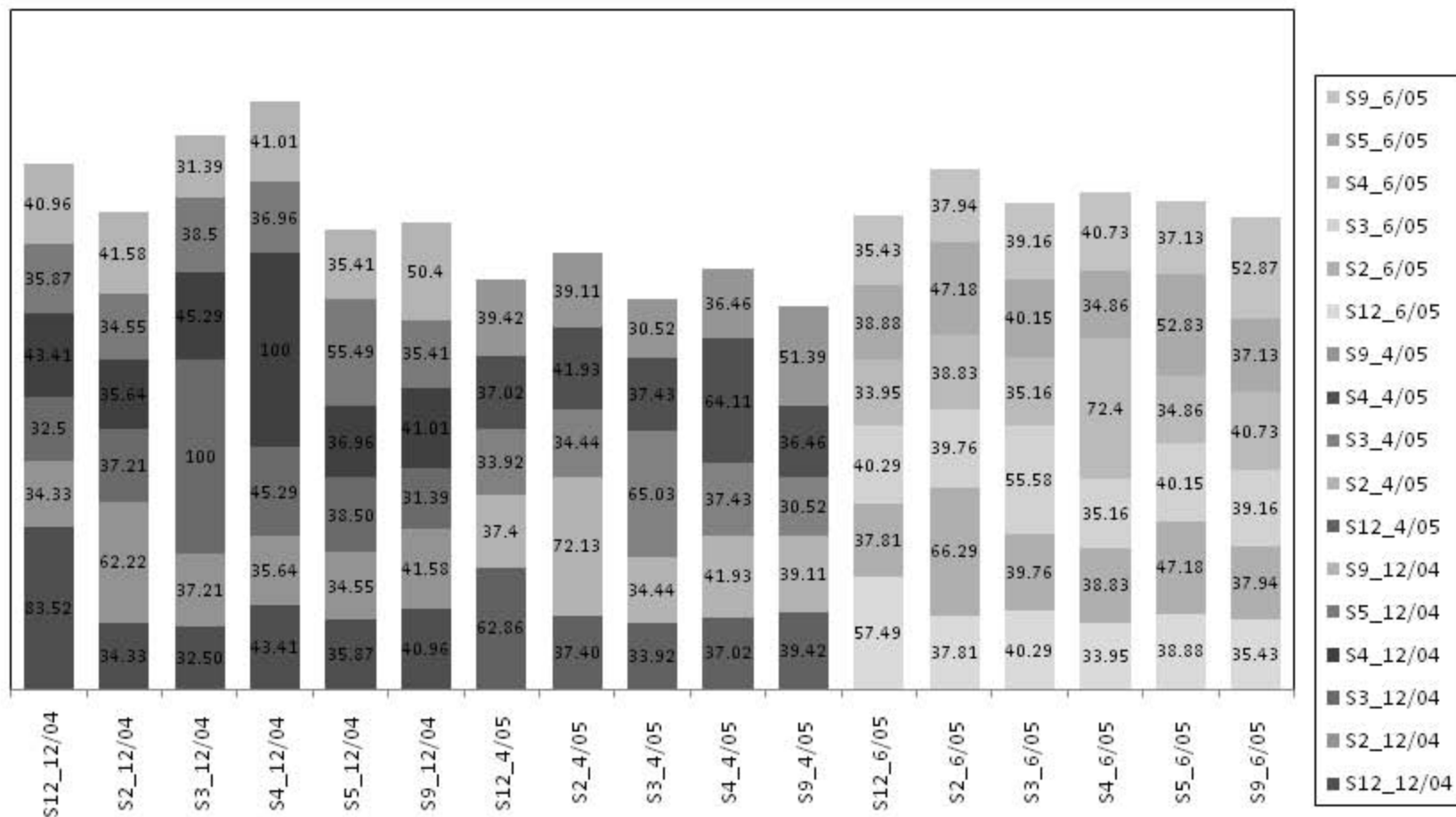

Sites

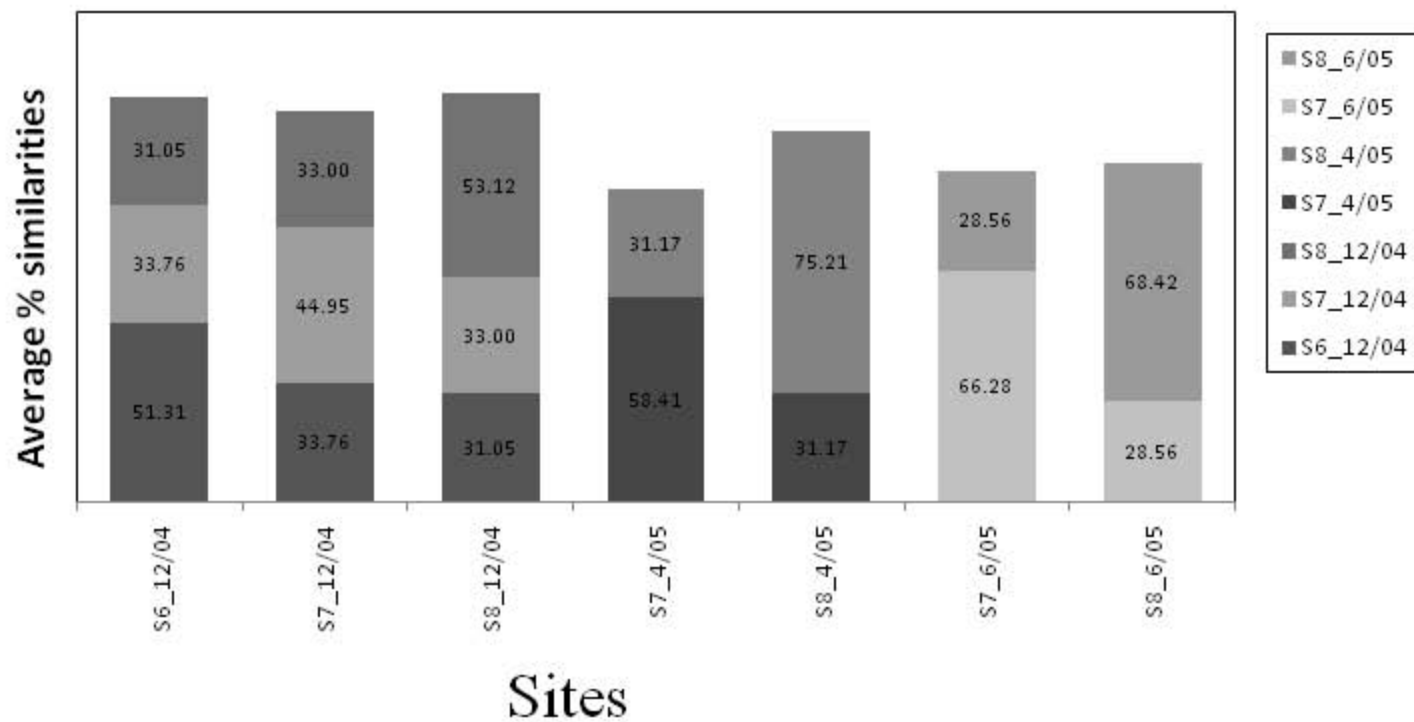

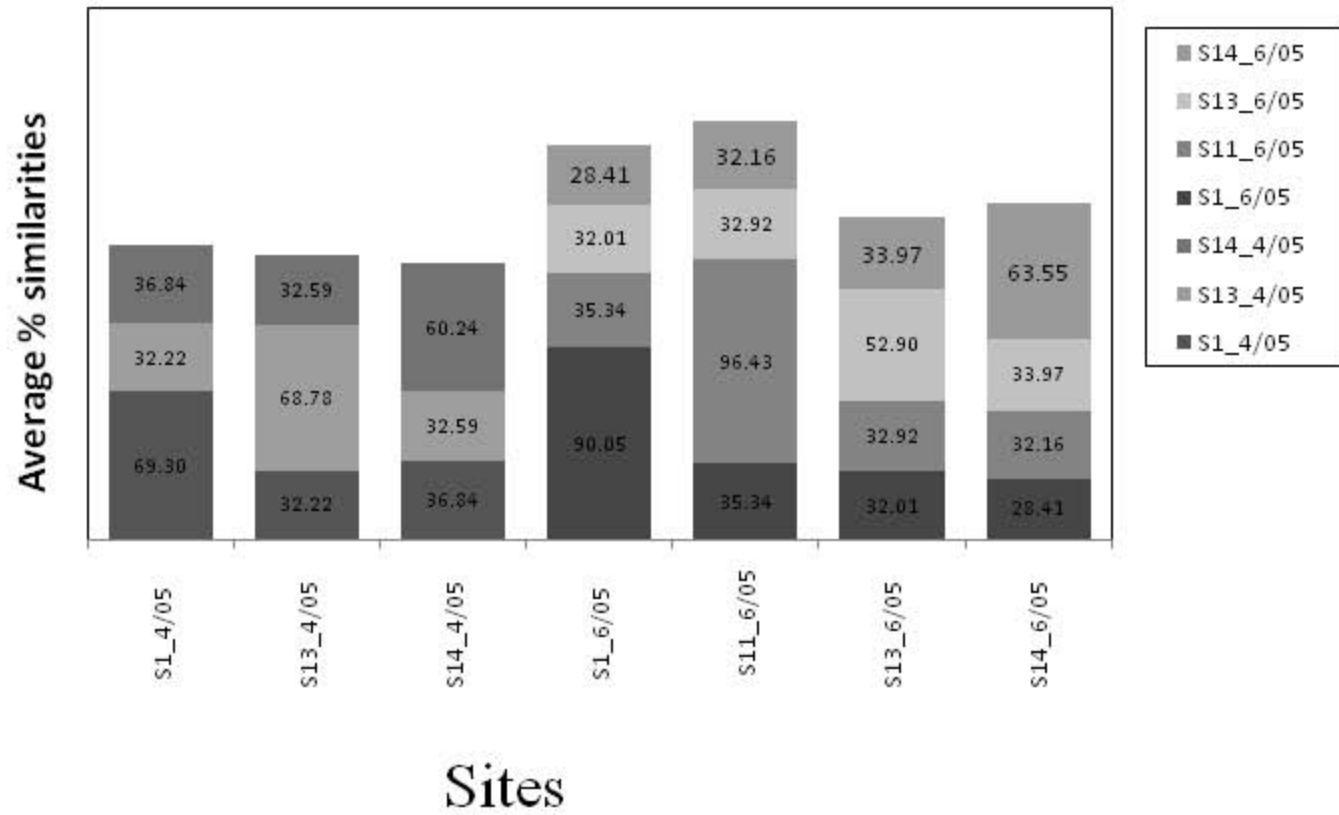

Average % similarities

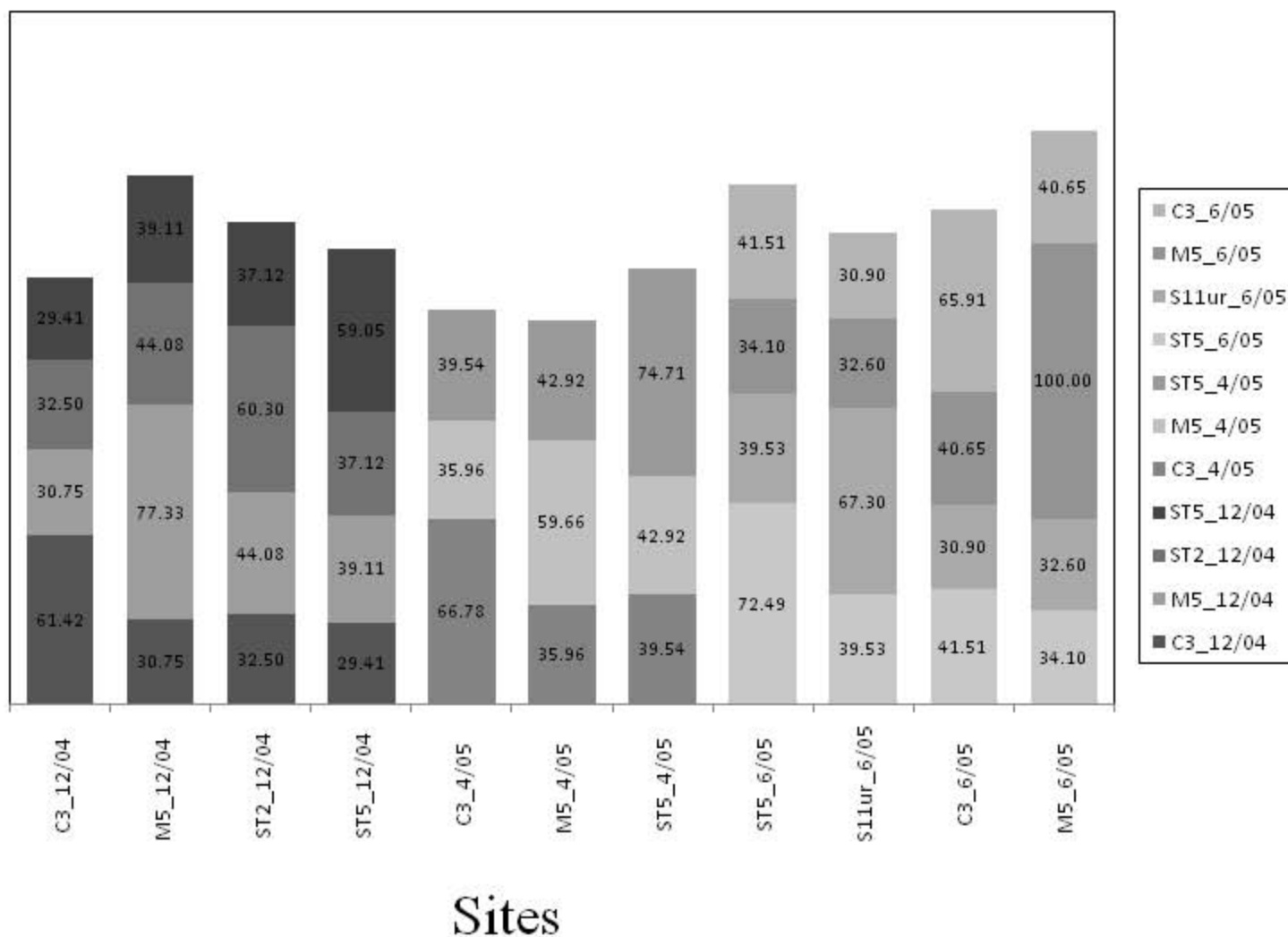

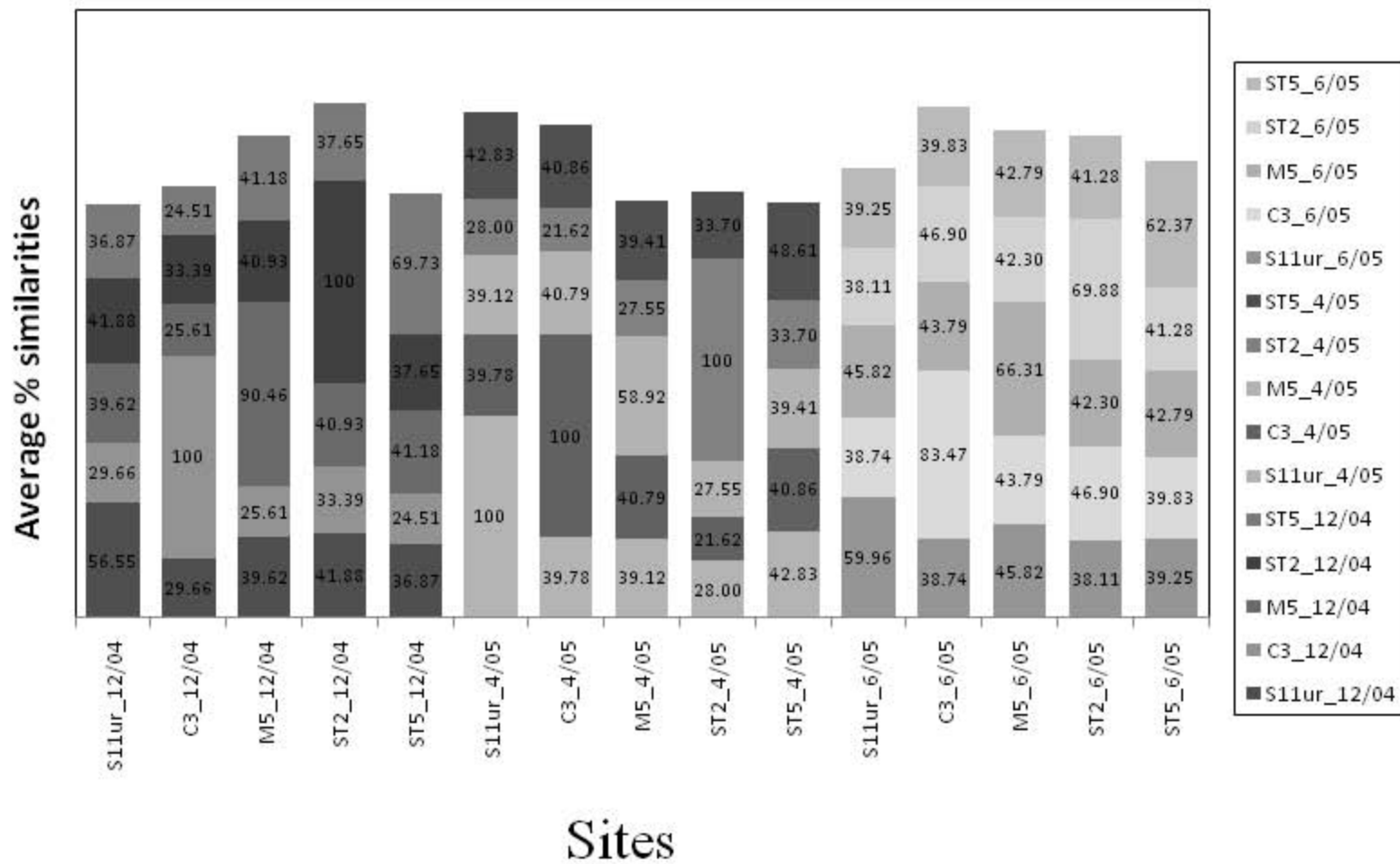

Supplement: Figure S1 — The pattern of diversity of E. coli isolates in the MSAR watershed: (a) soil from Chino creek, (b) water from Chino Creek, (c) water from Cypress channel, (d) water from WWTPs, (e) soil from Prado, and (f) water from the Prado park area. Sample names on the X axis are as shown in Figure 1 and Table 1 followed by dates that samples were collected from the different sites. (PDF) [file pone.0020819.s001.pdf]
