# Supplementary material for: Genetic Diversity and Antimicrobial Resistance of Escherichia coli from Human and Animal Sources Uncovers Multiple Resistances from Human Sources
Source: PLoS One. 2011 Jun 8;6(6):e20819. doi: 10.1371/journal.pone.0020819 (PMC3110821; doi:10.1371/journal.pone.0020819)
Supplement: Figure S2 — Multiply antimicrobial resistant E. coli isolates. The bars in Figure 5a as an example shows; S12-s, S2-s, and S3-s showed each site with five, five, and nine isolates, respectively, with multiple antimicrobial resistance phenotypes. S after the site names in Table 1 indicates the sample was taken from sediment. (PDF) [file pone.0020819.s002.pdf]

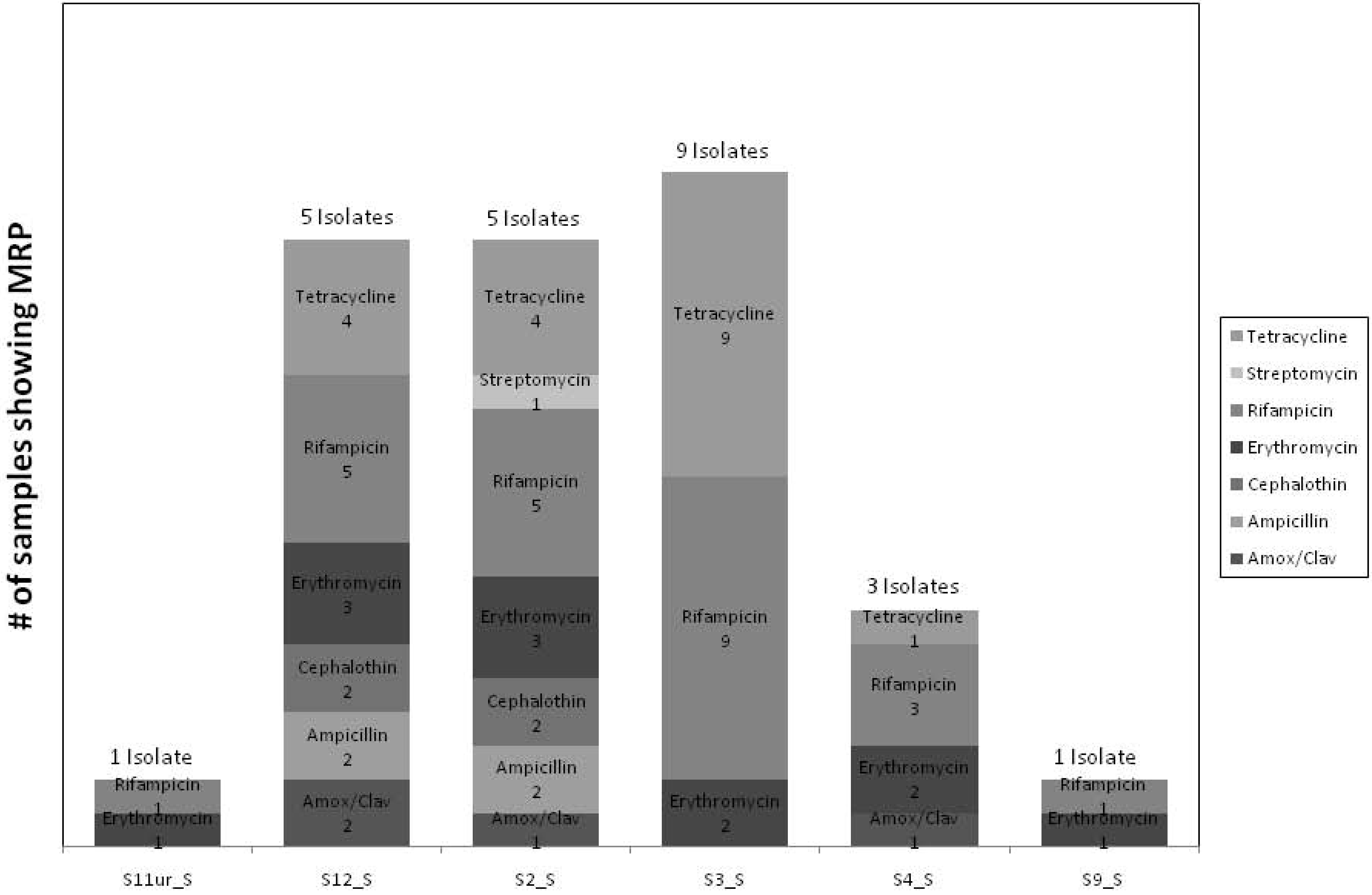

# of samples showing MRP
1 Isolate
Rifampicin 1
Erythromycin 1
5 Isolates
Tetracycline 4
Rifampicin 5
Erythromycin 3
Cephalothin 2
Ampicillin 2
Amox/Clav 2
5 Isolates
Tetracycline 4
Streptomycin 1
Rifampicin 5
Erythromycin 3
Cephalothin 2
Ampicillin 2
Amox/Clav 1
9 Isolates
Tetracycline 9
Rifampicin 9
Erythromycin 2
3 Isolates
Tetracycline 1
Rifampicin 3
Erythromycin 2
Amox/Clav 1
1 Isolate
Rifampicin 1
Erythromycin 1
S11ur_S
S12_S
S2_S
S3_S
S4_S
S9_S
Tetracycline
Streptomycin
Rifampicin
Erythromycin
Cephalothin
Ampicillin
Amox/Clav

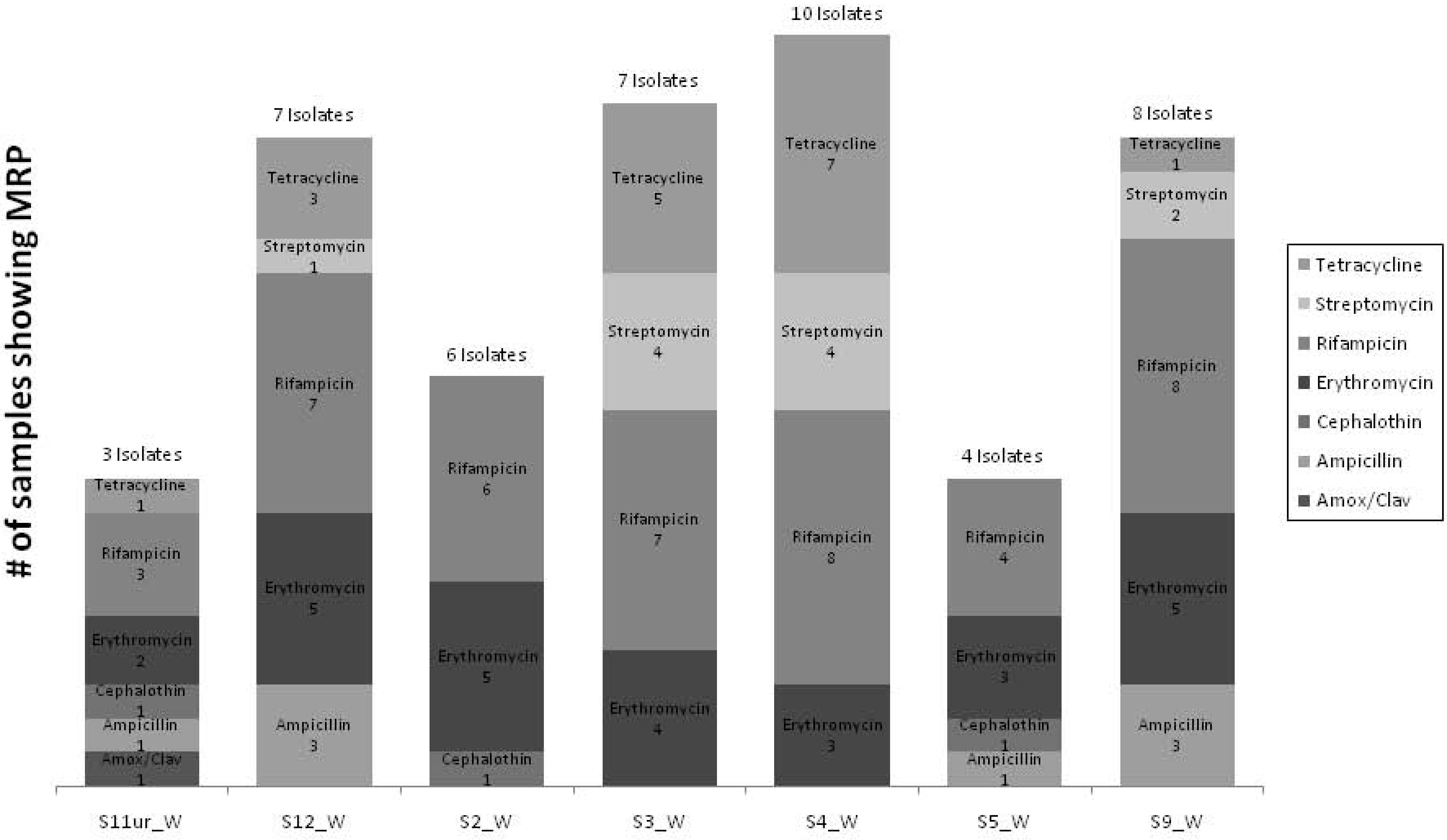

# of samples showing MRP
3 Isolates
7 Isolates
6 Isolates
7 Isolates
10 Isolates
4 Isolates
8 Isolates
Tetracycline 1
Rifampicin 3
Erythromycin 2
Cephalothin 1
Ampicillin 1
Amox/Clav 1
Tetracycline 3
Streptomycin 1
Rifampicin 7
Erythromycin 5
Ampicillin 3
Rifampicin 6
Erythromycin 5
Cephalothin 1
Tetracycline 5
Streptomycin 4
Rifampicin 7
Erythromycin 4
Tetracycline 7
Streptomycin 4
Rifampicin 8
Erythromycin 3
Rifampicin 4
Erythromycin 3
Cephalothin 1
Ampicillin 1
Tetracycline 1
Streptomycin 2
Rifampicin 8
Erythromycin 5
Ampicillin 3
S11ur_W
S12_W
S2_W
S3_W
S4_W
S5_W
S9_W
Tetracycline
Streptomycin
Rifampicin
Erythromycin
Cephalothin
Ampicillin
Amox/Clav

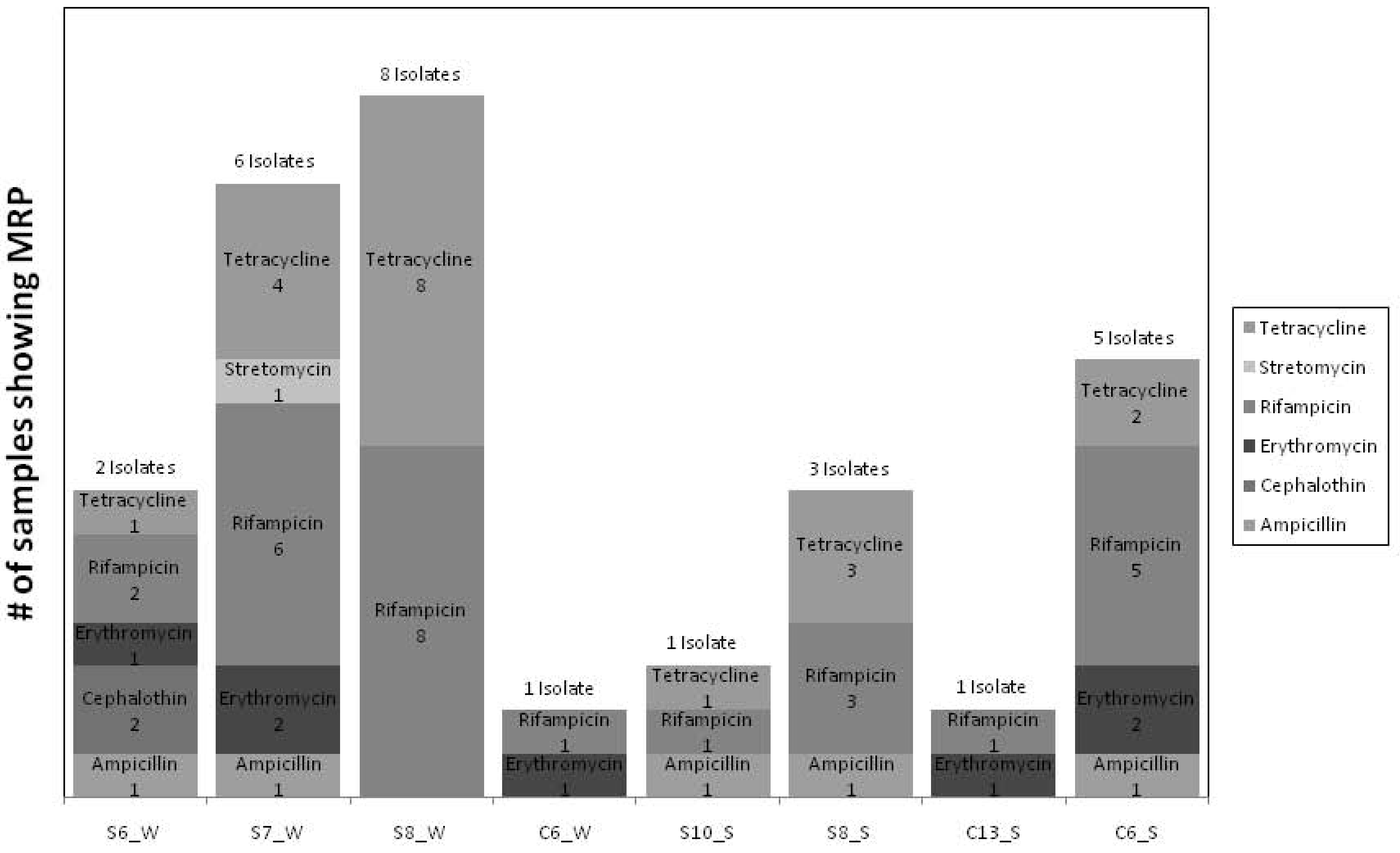

# of samples showing MRP
2 Isolates
Tetracycline 1
Rifampicin 2
Erythromycin 1
Cephalothin 2
Ampicillin 1
6 Isolates
Tetracycline 4
Stretomycin 1
Rifampicin 6
Erythromycin 2
Ampicillin 1
8 Isolates
Tetracycline 8
Rifampicin 8
1 Isolate
Rifampicin 1
Erythromycin 1
1 Isolate
Tetracycline 1
Rifampicin 1
Ampicillin 1
3 Isolates
Tetracycline 3
Rifampicin 3
Ampicillin 1
1 Isolate
Rifampicin 1
Erythromycin 1
5 Isolates
Tetracycline 2
Rifampicin 5
Erythromycin 2
Ampicillin 1
S6_W
S7_W
S8_W
C6_W
S10_S
S8_S
C13_S
C6_S
Tetracycline
Stretomycin
Rifampicin
Erythromycin
Cephalothin
Ampicillin

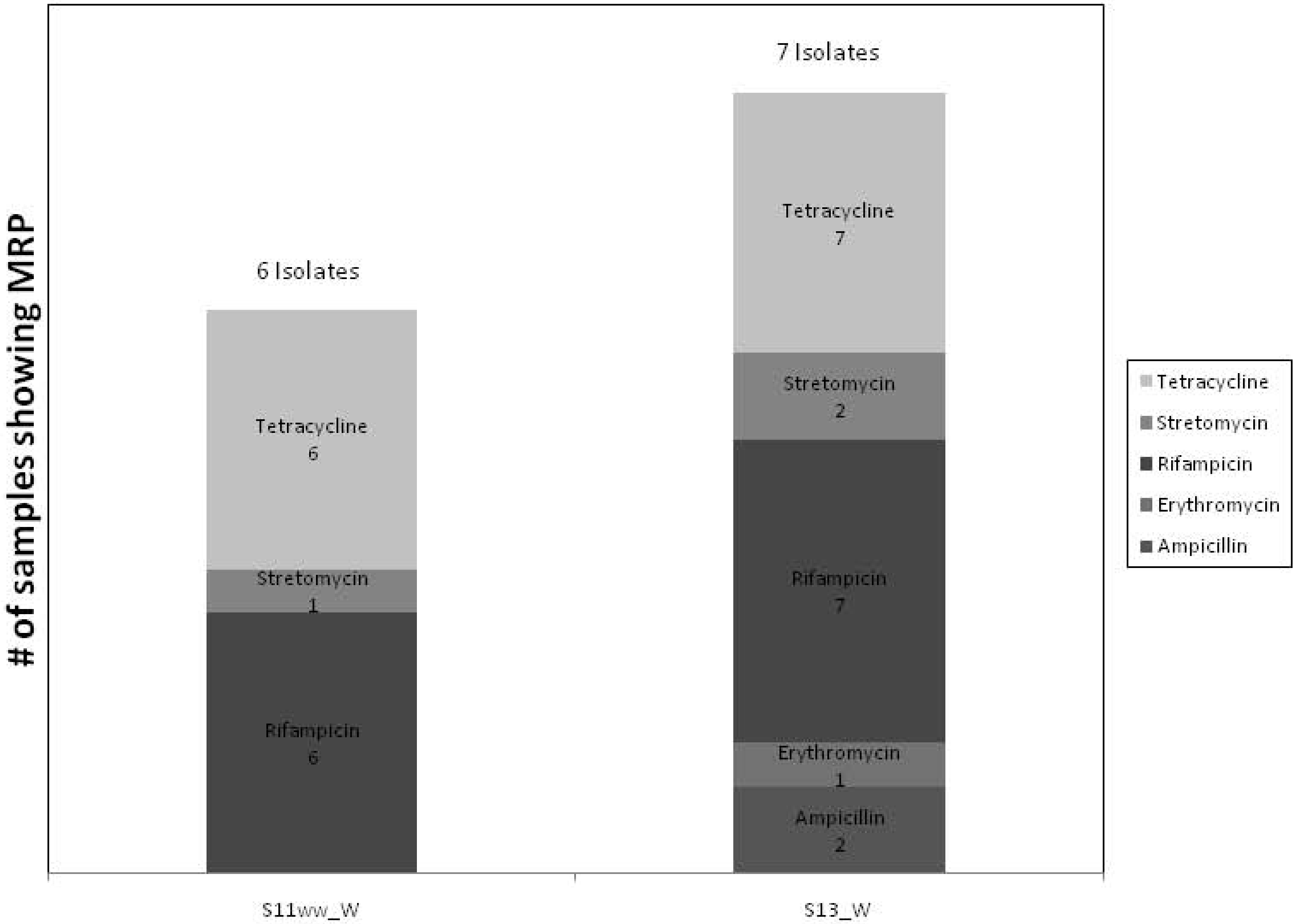

# of samples showing MRP
6 Isolates
7 Isolates
Tetracycline
6
Stretomycin
1
Rifampicin
6
Tetracycline
7
Stretomycin
2
Rifampicin
7
Erythromycin
1
Ampicillin
2
S11ww_W
S13_W
Tetracycline
Stretomycin
Rifampicin
Erythromycin
Ampicillin

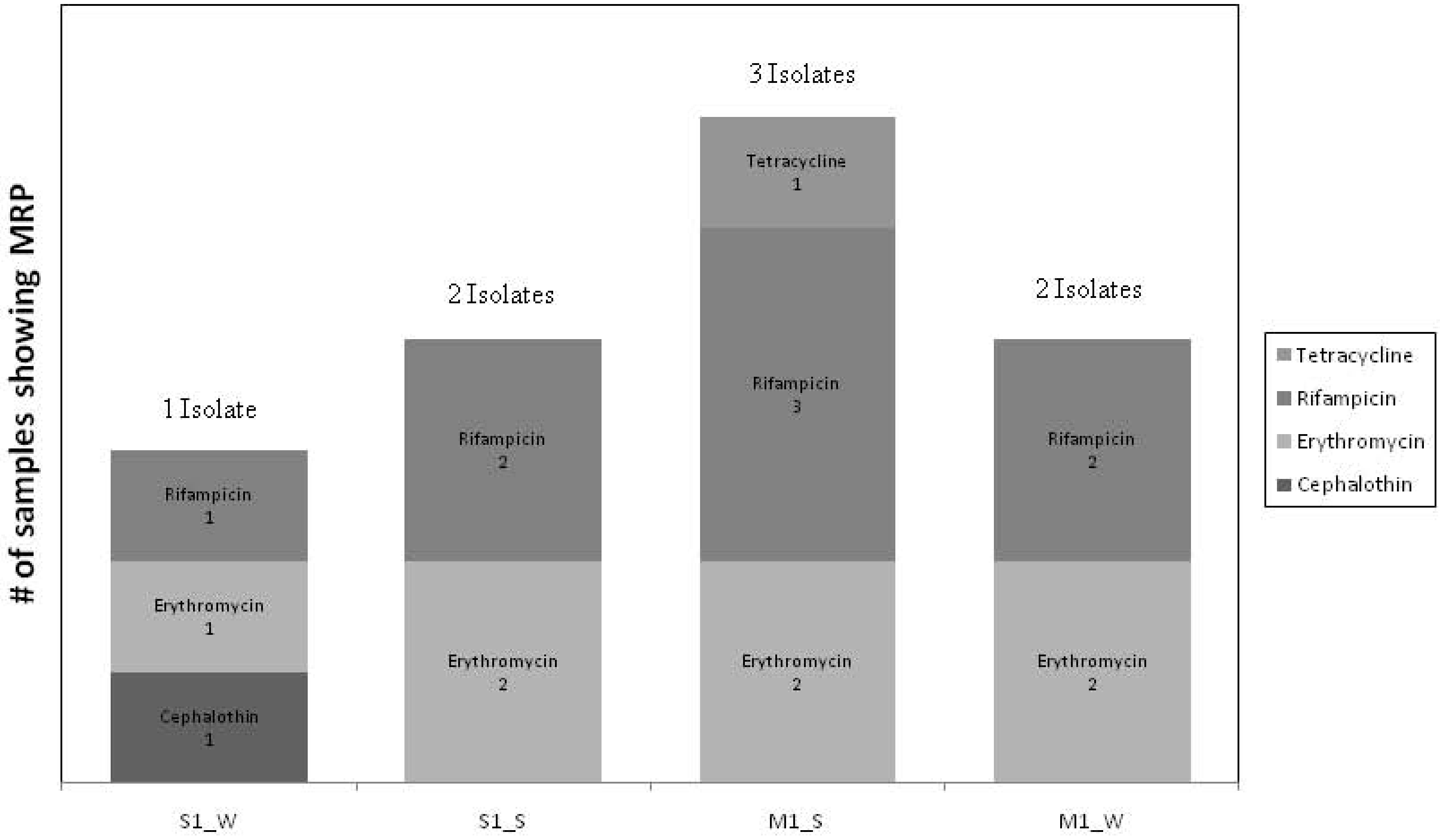

# of samples showing MRP
1 Isolate
2 Isolates
3 Isolates
2 Isolates
Cephalothin 1
Erythromycin 1
Rifampicin 1
Erythromycin 2
Rifampicin 2
Erythromycin 2
Rifampicin 3
Tetracycline 1
Erythromycin 2
Rifampicin 2
Tetracycline
Rifampicin
Erythromycin
Cephalothin
S1_W
S1_S
M1_S
M1_W

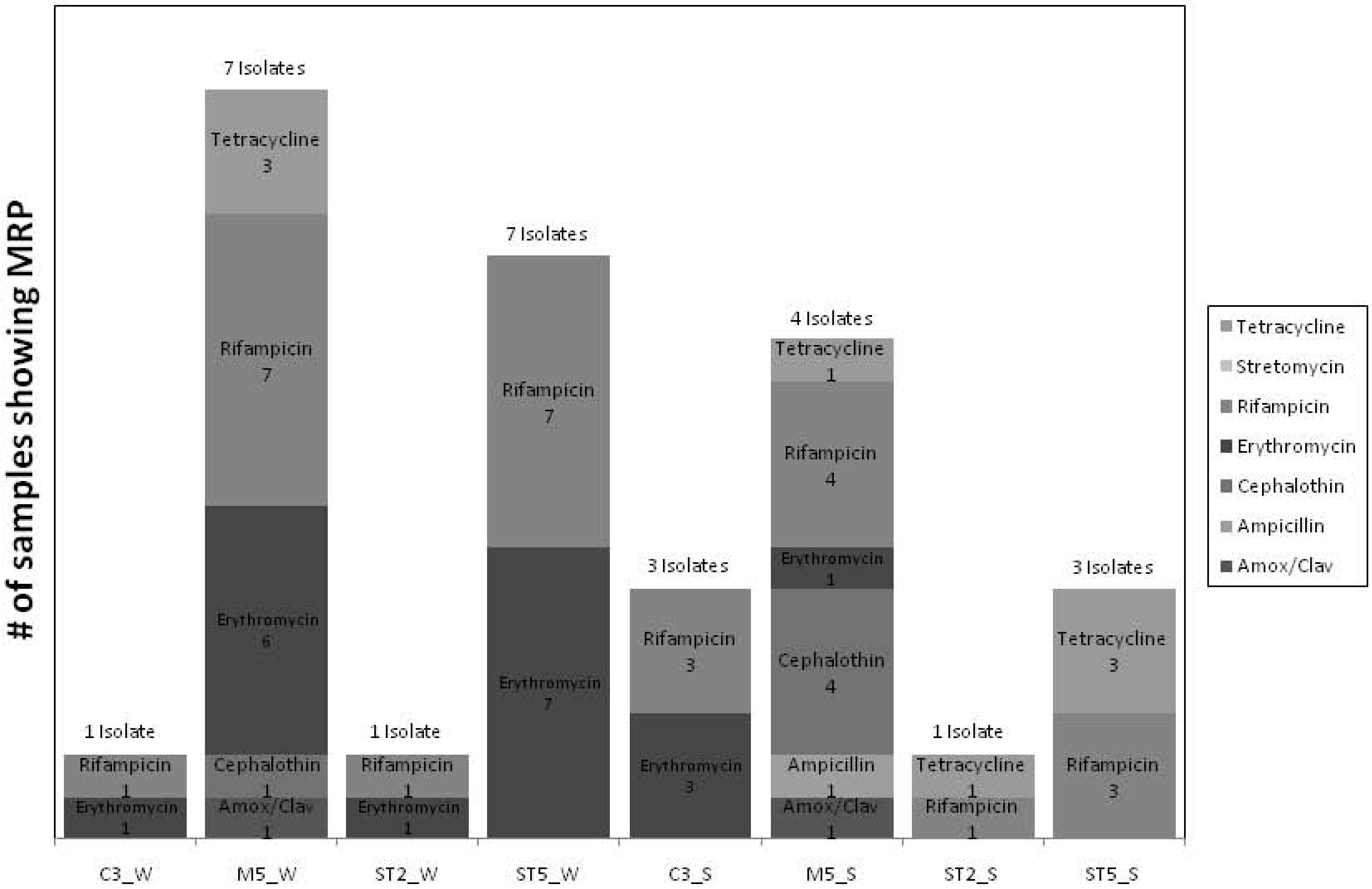

# of samples showing MRP
1 Isolate
Rifampicin 1
Erythromycin 1
7 Isolates
Tetracycline 3
Rifampicin 7
Erythromycin 6
Cephalothin 1
Amox/Clav 1
1 Isolate
Rifampicin 1
Erythromycin 1
7 Isolates
Rifampicin 7
Erythromycin 7
3 Isolates
Rifampicin 3
Erythromycin 3
4 Isolates
Tetracycline 1
Rifampicin 4
Erythromycin 1
Cephalothin 4
Ampicillin 1
Amox/Clav 1
1 Isolate
Tetracycline 1
Rifampicin 1
3 Isolates
Tetracycline 3
Rifampicin 3
C3_W
M5_W
ST2_W
ST5_W
C3_S
M5_S
ST2_S
ST5_S
Tetracycline
Stretomycin
Rifampicin
Erythromycin
Cephalothin
Ampicillin
Amox/Clav
